# Calculating the motion of highly confined, arbitrary-shaped particles in Hele–Shaw channels

**DOI:** 10.1007/s10404-018-2092-y

**Published:** 2018-07-12

**Authors:** Bram Bet, Rumen Georgiev, William Uspal, Huseyin Burak Eral, René van Roij, Sela Samin

**Affiliations:** 1 Center for Extreme Matter and Emergent Phenomena, Institute for Theoretical Physics, Utrecht University, Princetonplein 5, 3584 CC Utrecht, The Netherlands; 2 Process and Energy Department, Delft University of Technology, 2628 CD Delft, The Netherlands; 3 Van’t Hoff Laboratory for Physical and Colloid Chemistry, Debye Institute for Nanomaterials Science, Utrecht University, 3508 TB Utrecht, The Netherlands; 4 Max Planck Institute for Intelligent Systems, Heisenbergstr. 3, 70569 Stuttgart, Germany; 5 IV. Institut für Theoretische Physik, Universitt Stuttgart, Pfaffenwaldring 57, 70569 Stuttgart, Germany

## Abstract

We combine theory and numerical calculations to accurately predict the motion of anisotropic particles in shallow microfluidic channels, in which the particles are strongly confined in the vertical direction. We formulate an effective quasi-two-dimensional description of the Stokes flow around the particle via the Brinkman equation, which can be solved in a time that is two orders of magnitude faster than the three-dimensional problem. The computational speedup enables us to calculate the full trajectories of particles in the channel. To validate our scheme, we study the motion of dumbbell-shaped particles that are produced in a microfluidic channel using ‘continuous-flow lithography’. Contrary to what was reported in earlier work (Uspal et al. in Nat Commun 4:2666, 2013), we find that the reorientation time of a dumbbell particle in an external flow exhibits a minimum as a function of its disk size ratio. This finding is in excellent agreement with new experiments, thus confirming the predictive power of our scheme.

**Electronic supplementary material:**

The online version of this article (10.1007/s10404-018-2092-y) contains supplementary material, which is available to authorized users.

## Introduction

Microfluidic devices offer many applications, such as flow cytometry (Oakey et al. 2010; Wang et al. 2007; Mao et al. 2009), separation of cells (Gossett et al. 2010), DNA sequencing (Tewhey et al. 2009), or blood cell analysis (Toner and Irimia 2005). In many applications, it is of paramount importance to control the position of the immersed particles. Often, focusing of particles in the channel is achieved by external fields or by flows induced by the channel geometry (Xuan et al. 2010), while particle separation or sorting can also be realised by tuning electric fields (Jeon et al. 2016; Wang et al. 2007) or the channel geometry (Gossett et al. 2010; Sajeesh and Sen 2014; Pamme 2007; Zeming et al. 2013; Li et al. 2014). Alternatively, the shape of the particle itself offers a different route to manoeuvring the particles in the channel (Masaeli et al. 2012). The hydrodynamics of various particles in strong confinement has been extensively studied; for instance, the motion of confined droplets (Beatus et al. 2012; Shen et al. 2014), (connected) disks (Uspal and Doyle 2012; Uspal et al. 2013), and fibers (Berthet et al. 2013; Nagel et al. 2018). However, as tunability increases with the complexity of the particle shapes, theoretical arguments on the basis of simplified geometries might fall short of accurately describing the motion of particles. In addition, advances in versatile particle synthesis techniques, such as continuous-flow lithography (Dendukuri et al. 2006), make an infinite variety of quasi-two-dimensional shapes experimentally accessible.

**Fig. 1:**
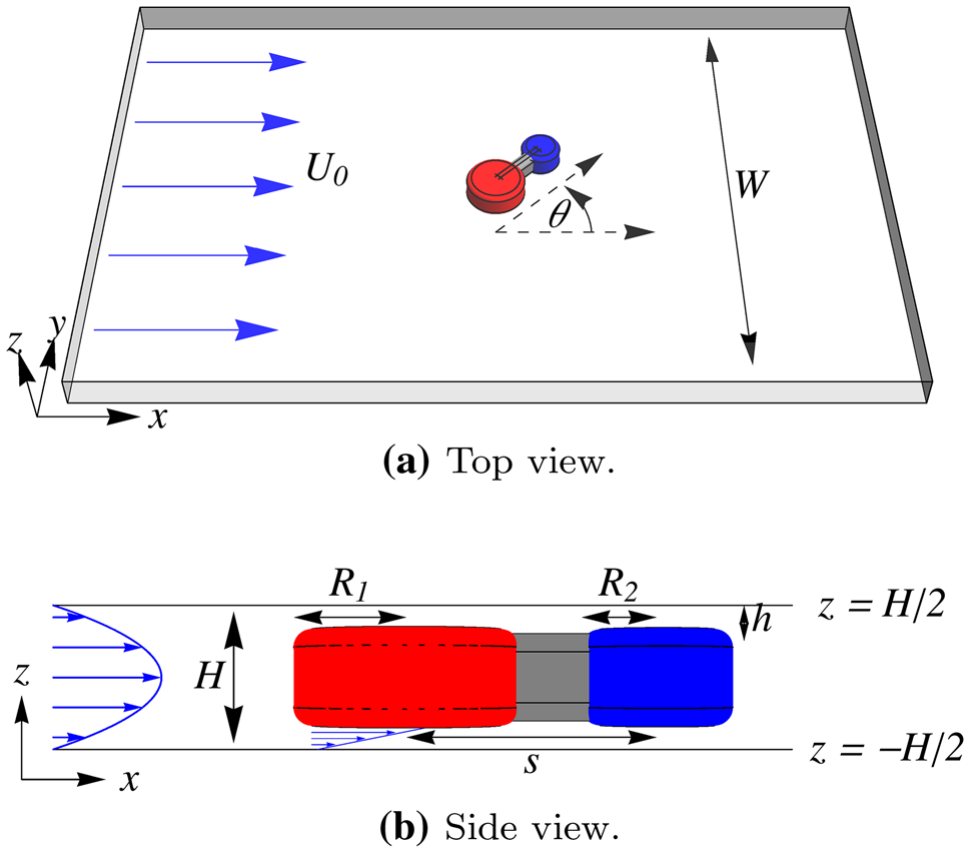
Top view **a** and side view **b** of the geometry and the Cartesian frame. An external flow $\varvec{U}_0$ with parabolic profile is imposed through a channel of length *L*, width *W*, and height *H*, containing a dumbbell particle with radii $R_1$ and $R_2$ at a center-to-center distance *s*, with height $H-2 h$, such that the height of the gaps between the particle and the top and bottom walls is given by *h*. In these gaps, the flow profile is approximately that of a simple shear flow. The orientation of the long axes of the dumbbell with respect to the external flow $\varvec{U}_0$ is denoted by $\theta$

Inspired by these advances, we develop, in this work, a method to calculate the two-dimensional motion of confined particles in microfluidic channels that can handle particles of any given quasi-two-dimensional shape. Here, we combine finite-element calculations with a simple approach for the particle motion to solve the full hydrodynamic equations at hand, either in full detail in the three-dimensional geometry or in an effective two-dimensional description. Our method is validated by comparison of the three- and two-dimensional results with analytical calculations. Next, we apply our method to study dumbbell-shaped particles in Hele–Shaw channels, reproducing new experimental results accurately, without any adjustable parameters.

## Theory and numerical methods

### Hydrodynamic equations and equations of motion

We consider a rigid particle, of arbitrary shape, at position $\varvec{r}_{\text {p}} = (x_{\text {p}},y_{\text {p}},z_{\text {p}})$, which is immersed in a fluid that is driven through a shallow microfluidic channel by the application of pressure at the channel inlet, see the illustration in Fig. 1. Let us assume that this particle is strongly confined in the *z*-direction, i.e., the particle is separated from the top and bottom walls by a small gap of height *h* that is much smaller than the channel height *H*. Such a particle can be produced, for example, using ‘continuous-flow lithography’ (Dendukuri et al. 2006). In this method, the fluid in the channel is a prepolymer solution, where particles are ‘printed’ by crosslinking the oligomers by pulses of UV light, which is applied through a photolithographic mask. In this way, the particle shape is defined in the *xy*-plane by the shape of the mask (which can be of any desired shape), while it is extruded in the *z*-direction to a height that is comparable to the channel height, such that $h \ll H$.

The microfluidic channel under consideration here (see Appendix 1) has a height $H = {30}\,\upmu$m and a much larger width $W = {500}\,\upmu$m, while the length *L* of the channel is of the order of 1 cm, which can be considered infinite for our analysis. The fluid is driven through the channel at an average velocity $U_0$, which is of the order of ${50}\,\upmu {\text {m}}\,{\text {s}}^{-1}$. Using the hydraulic diameter $D_H=2HW/(H+W)$ as the characteristic length scale, we find that the Reynolds number is $Re=\rho U_0 D_H/\eta = 10^{-4} - 10^{-5}$ for a typical oligomer solution with viscosity $\eta$ and density $\rho$. Therefore, the flow is well described by the Stokes equation (Kim and Karrila 1991; Happel and Brenner 2012; Leal 2007):

1 $$\begin{aligned} - \nabla p + \eta \nabla ^2 \varvec{u} =0, \quad \nabla \cdot \varvec{u} =0, \end{aligned}$$

where $\varvec{u}(\varvec{r})$ and $p(\varvec{r})$ are the fluid velocity and pressure at position $\varvec{r},$ respectively, and $\eta$ denotes the fluid viscosity. We supplement the Stokes equation with no-slip boundary conditions on the (stationary) channel walls and the (moving) particle surface $S_{\text {p}}$:

2 $$\begin{aligned} \varvec{u}\big |_{\text {walls}} = 0, \quad \varvec{u}\big |_{\varvec{r} \in S_{\text {p}}} = \varvec{U}_{\text {p}} + \varvec{\omega }_{\text {p}} \times (\varvec{r}-\varvec{r}_{\text {p}}), \end{aligned}$$

where $\varvec{U}_{\text {p}}$ and $\varvec{\omega }_{\text {p}}$ denote the particle velocity and angular velocity, respectively. At the inlet of the channel, we impose a uniform incoming Hele–Shaw flow:

3 $$\begin{aligned} \varvec{u}\big |_{\text {inlet}} = \varvec{U}_0(\varvec{r}) = \frac{3}{2}\left( 1-\frac{4 z^2}{H^2}\right) (U_0,0,0), \end{aligned}$$

which is the analytic solution for the flow between two infinite parallel plates driven by a constant pressure difference. Here, $z \in [-H/2,H/2]$ and $U_0$ is the average velocity in the *x*-direction. Note that this boundary condition neglects the no-slip condition on the sidewalls, i.e., the prescribed form (3) does not vanish at the side walls at $y = \pm W/2$. Therefore, we take into account a finite ‘entrance length’ at the inlet after which the flow is fully developed downstream in the channel. Finally, we impose a zero-pressure boundary condition at the outlet, such that the pressure difference between inlet and outlet is precisely driving the flow field given by Eq. (3), i.e., $U_0 = - H^2 \nabla p /(12 \eta )$. The influence of the no-slip side walls on this pressure drop is assumed to be negligible. The Stokes equation (1), together with these boundary conditions, forms a closed set of equations that we solve numerically by a finite-element scheme (see Sect. 2.3).

The fluid in the channel surrounding the particle exerts a hydrodynamic force $\varvec{F}$ and torque $\varvec{T}$ on the particle, which is calculated by integrating the hydrodynamic stress tensor, $\sigma _{ij} = -p \delta _{ij} + \eta (\partial _i u_j + \partial _j u_i)$, over the particle surface:

4 $$\begin{aligned} \varvec{F} = \int _{S_{\text {p}}}{\text {d}}S \ \sigma \cdot \varvec{n}, \quad \varvec{T} = \int _{S_{\text {p}}}{\text {d}}S \ (\varvec{r}- \varvec{r}_{\text {p}}) \times (\sigma \cdot \varvec{n}). \end{aligned}$$

Due to the linearity of the Stokes equation (1), we can write the solution $(p,\varvec{u})$ of Eq. (1) with boundary conditions (2) and (3) as a superposition of two solutions (Happel and Brenner 2012): $\varvec{u} = \varvec{u}_0 + \varvec{u}'$ and $p = p_0 + p'$, where $(p_0,\varvec{u}_0)$ and $(p',\varvec{u}')$ are solutions to the Stokes equation (1) with boundary conditions:

5 $$\begin{aligned} \varvec{u}_0\big |_{\text {walls}}&= 0, \quad \varvec{u}_0\big |_{\text {inlet}} = \varvec{U}_0(\varvec{r}), \quad \varvec{u}_0\big |_{\varvec{r} \in S_{\text {p}}} = 0; \end{aligned}$$

6 $$\begin{aligned} \varvec{u}'\big |_{\text {walls}}&= 0, \quad \varvec{u}'\big |_{\text {inlet}} = 0, \end{aligned}$$

7 $$\begin{aligned} \varvec{u}'\big |_{\varvec{r} \in S_{\text {p}}}&= \varvec{U}_{\text {p}} + \varvec{\omega }_{\text {p}} \times (\varvec{r}-\varvec{r}_{\text {p}}) , \end{aligned}$$

that is, $\varvec{u}_0$ is the solution where the particle is fixed subject to the imposed external flow, and $\varvec{u}'$ the solution where the particle moves through the channel without an imposed flow. The stress tensor also splits accordingly, $\sigma = \sigma _0 + \sigma '$, such that we find that the forces and torque on the particle are written as $\varvec{F} = \varvec{F}_0 + \varvec{F}'$ and $\varvec{T}= \varvec{T}_0 + \varvec{T}'$, which are calculated from Eq. (4) by replacing $\sigma$ with $\sigma _0$ or $\sigma '$.

To proceed, we can again use the linearity of the Stokes equation (1) to derive that the force $\varvec{F}'$ and torque $T'$ depend linearly on each component of the particle (angular) velocity via a $6 \times 6$ resistance tensor $\mathcal {R}$ as (Happel and Brenner 2012; Brenner 1963, 1964):

8 $$\begin{aligned} \left( \begin{array}{c}\varvec{F}' \\ \varvec{T}'\end{array}\right) = - \eta {\mathcal {R}}\left( \begin{array}{c}\varvec{U}_{\text {p}} \\ \omega _{\text {p}}\end{array}\right) . \end{aligned}$$

Due to the overdamped nature of the system, the (hydrodynamic) force and torque on the particle vanish in the absence of external forces on the particle. Therefore, after summing up the force contributions from the solutions $\varvec{u}_0$ and $\varvec{u}'$, we find that the particle must obey the equation of motion:

9 $$\begin{aligned} \left( \begin{array}{c}\varvec{F} \\ \varvec{T}\end{array}\right) = - \eta {\mathcal {R}}\left( \begin{array}{c}\varvec{U}_{\text {p}} \\ \varvec{\omega }_{\text {p}}\end{array}\right) +\left( \begin{array}{c}\varvec{F}_0 \\ \varvec{T}_0 \end{array}\right) = \left( \begin{array}{c}\varvec{0} \\ \varvec{0}\end{array}\right) , \end{aligned}$$

with $\varvec{0} = (0,0,0)$. Thus, once ${\mathcal {R}}, \varvec{F}_0$ and $\varvec{T}_0$ are determined, either analytically or numerically, the equations of motion (9) can be solved for the particle velocity and angular velocity as follows:

10 $$\begin{aligned} \left( \begin{array}{c}\varvec{U}_{\text {p}} \\ \varvec{\omega }_{\text {p}}\end{array}\right) = \frac{1}{\eta } {\mathcal {R}}^{-1} \left( \begin{array}{c}\varvec{F}_0 \\ \varvec{T}_0 \end{array}\right) . \end{aligned}$$

Notice that ${\mathcal {R}}, \varvec{F}_0$ and $\varvec{T}_0$ depend on the position and orientation of the particle, such that Eq. (10) only determines the force- and torque-free (angular) velocity for that specific particle position and velocity. The position dependence of $\mathcal {R}$ is related to the effects of the side walls; in the case of an infinite slit, the tensor $\mathcal {R}$ will only depend on the particle geometry and its orientation (with respect to the imposed external flow).

In this work, due to the strong confinement in the vertical direction as well as the mirror symmetry in the $z=0$ plane of the problem at hand, the movement of the particle is restricted to the two-dimensional mid-plane of the channel at $z=0$, thereby reducing the number of degrees of freedom to three: $\varvec{U}_{\text {p}} = (U_{{\text {p}},x},U_{{\text {p}},y},0)$ and $\varvec{\omega }_{\text {p}} = (0,0,\omega _{\text {p}})$, and similarly $\varvec{F} = (F_x , F_y ,0)$ and $\varvec{T} = (0 , 0, T).$^1^ Moreover, the particle position is determined by the coordinates $(x_{\text {p}} , y_{\text {p}})$, while its orientation is described by a single angle $\theta$, as illustrated in Fig. 1. Finally, the resistance tensor $\mathcal {R}$ reduces to a $3 \times 3$ tensor, which is obtained from the relevant components of the original $6 \times 6$ resistance tensor. In some cases, symmetry arguments may be invoked to reduce the number of degrees of freedom even further, e.g., mirror symmetric particles that are aligned with the imposed external flow, as we shall see below.

Using a finite-element scheme, we can numerically solve the flow field $\varvec{u}$ for any imposed velocity and angular velocity, and from these solutions, we can obtain the resistance tensor $\mathcal {R}$, the force $\varvec{F}_0$, and the torque $T_0$, to subsequently obtain the force- and torque-free velocities from Eq. (10). By repeating this at the new particle position in the next time step, it is, in principle, possible to integrate the complete particle motion.

### Brinkman equation

A three-dimensional finite-element calculation is able to resolve the flow in the channel. However, due to a separation of length scales, $h \ll W$, the finite-element mesh needs to be chosen very fine at certain places, causing the calculations to be computationally costly, and eventually prohibitive for the purpose of integrating the particle motion. To circumvent this problem, we resort to an effective 2D-description of the system via the Brinkman equation (Brinkman 1949; Uspal and Doyle 2014).

Far enough from the side walls^2^ and the particle, the flow field is well described by Hele–Shaw flow:

11 $$\begin{aligned} \varvec{u}(x,y,z) = \frac{3}{2}\bar{\varvec{u}} (x,y) \left( 1 - \frac{ 4 z^2}{H^2} \right) , \end{aligned}$$

the pressure being independent of *z* to a good approximation: $p = p(x,y)$. We substitute this ansatz in the Stokes equation (1) and average over the channel height to find the Brinkman equation (Brinkman 1949; Uspal and Doyle 2014):

12 $$\begin{aligned} - \nabla _{\text {2D}} \bar{p} + \eta H \nabla _{\text {2D}}^2 \ \bar{\varvec{u}} - \frac{12 \eta }{ H} \bar{\varvec{u}} =0, \quad \nabla _{\text {2D}} \cdot \bar{\varvec{u}}=0, \end{aligned}$$

where the two-dimensional vector field $\bar{\varvec{u}} = (\bar{u}_x,\bar{u}_y)$ denotes the *z*-averaged value of the three-dimensional flow field $\varvec{u}$, and $\bar{p} = p H$ denotes the two-dimensional pressure field. Henceforth, we will denote the two-dimensional height-averaged quantities with an overbar. The boundary conditions that supplement Eq. (12) are accordingly:

13 $$\begin{aligned}&\bar{\varvec{u}}\big |_{\text {walls}} = 0, \quad \end{aligned}$$

14 $$\begin{aligned}&\bar{\varvec{u}}\big |_{\text {inlet}} = U_0 \hat{\varvec{x}}, \quad \end{aligned}$$

15 $$\begin{aligned}&\bar{\varvec{u}}\big |_{(x,y) \in \partial \bar{S}_{\text {p}}} = \varvec{U}_{\text {p}} + \omega _{\text {p}} (-(y-y_{\text {p}}),x-x_{\text {p}}), \end{aligned}$$

where $\varvec{r} = (x,y)$ and $\partial \bar{S}_{\text {p}}$ denotes the one-dimensional particle boundary of the projected particle surface $\bar{S}_{\text {p}}$. Notice that the walls in this situation are the projections of the sidewalls at $y = \pm W/2$. As before, $\varvec{U}_{\text {p}} = (U_{{\text {p}},x},U_{{\text {p}},y})$ and $\omega _{\text {p}}$ denote the particle velocity and angular velocity, respectively.

The solution $(\bar{p},\bar{\varvec{u}})$ of the Brinkman equation (12) defines a stress tensor:

16 $$\begin{aligned} \bar{\sigma }_{ij} = -\delta _{ij} \bar{p} + \eta H (\partial _i \bar{u}_j + \partial _j \bar{u}_i), \end{aligned}$$

which is integrated over the particle surface to find the hydrodynamic force and torque on the particle:

17 $$\begin{aligned} \varvec{F}_f&= \int _{\partial \bar{S}_{\text {p}}} {\text {d}}s \ \bar{\sigma } \cdot \varvec{n}, \quad \end{aligned}$$

18 $$\begin{aligned} T_f&= \int _{\partial \bar{S}_{\text {p}}} {\text {d}}s \ \bigg ( (x-x_{\text {p}})(\bar{\sigma } \cdot \varvec{n})_y - (y-y_{\text {p}})(\bar{\sigma } \cdot \varvec{n})_x \bigg ). \end{aligned}$$

The subscript ‘*f*’ indicates that this is the force and torque due to the surrounding two-dimensional fluid, and does not include the force and torque from the confining walls.

Similar to the Stokes equation, the Brinkman equation is linear in the fields $\bar{p}$ and $\bar{u}$. Using a similar decomposition as in the previous section, we prove explicitly in Appendix 3 that the force $\varvec{F}_f$ and torque $T_f$ admit a linear relation to the particle velocity and angular velocity in terms of a $3 \times 3$ resistance tensor $\mathcal {R}_f$:

19 $$\begin{aligned} \left( \begin{array}{c}\varvec{F} \\ T\end{array}\right) _f = -\eta {\mathcal {R}}_f \left( \begin{array}{c}\varvec{U}_{\text {p}} \\ \omega _{\text {p}} \end{array}\right) + \left( \begin{array}{c}\varvec{F}_0 \\ T_0 \end{array}\right) . \end{aligned}$$

In Appendix 3, it is shown that $\mathcal {R}_f$ is symmetric by employing a version of the Lorentz reciprocal theorem for solutions of the Brinkman equation, which is given in Appendix 2.

Since the fluid-filled gaps between the particle and the walls are not accounted in the Brinkman description, their contribution to the force and torque on the particle, which stem from the friction with the top and bottom wall, is missing. To obtain this contribution, we assume locally a simple shear flow in the narrow gaps between the particle and the confining walls. As a result, an area element ${\text {d}}S$ on either of the particle faces that moves with a velocity $\varvec{v}_S = \varvec{U}_{\text {p}} + \varvec{\omega }_{\text {p}} \times (\varvec{r}-\varvec{r}_{\text {p}})$ with respect to the wall will experience a friction force $\varvec{f}_S = - (\eta /h) \varvec{v}_S{\text {d}}S$. The total force $\varvec{F}_w$ on the particle due to the wall friction is then found by integrating over the particle-gap surface:

20 $$\begin{aligned} \varvec{F}_w = - \frac{2\eta }{h} \int _{\bar{S}_{\text {p}}} {\text {d}}S \ \left( \varvec{U}_{\text {p}} + \varvec{\omega }_{\text {p}} \times (\varvec{r}-\varvec{r}_{\text {p}}) \right) , \end{aligned}$$

where the factor 2 is due to the two gaps. The area element *dS* also generates a torque $\varvec{r} \times \varvec{f}_S$, which can be integrated to find the frictional torque on the particle due to the walls:

21 $$\begin{aligned} \varvec{T}_w = - \frac{2\eta }{h} \int _{\bar{S}_{\text {p}}} {\text {d}}S \ \bigg ((\varvec{r}-\varvec{r}_{\text {p}}) \times \left( \varvec{U}_{\text {p}} + \varvec{\omega }_{\text {p}} \times (\varvec{r}-\varvec{r}_{\text {p}}) \right) \bigg ). \end{aligned}$$

As before, the linear dependence of Eqs. (20) and (21) in the particle velocities leads to

22 $$\begin{aligned} \left( \begin{array}{c}\varvec{F} \\ T\end{array}\right) _w = -\eta {\mathcal {R}}_w \left( \begin{array}{c}\varvec{U}_{\text {p}} \\ \omega _{\text {p}} \end{array}\right) , \end{aligned}$$

where the components of the $3 \times 3$ wall resistance tensor $\mathcal {R}_w$ are calculated from (20) and (21) to be

23 $$\begin{aligned} {\mathcal {R}}_{w,11}&= {\mathcal {R}}_{w,22} = \frac{2}{h} \int _S {\text {d}}S; \end{aligned}$$

24 $$\begin{aligned} {\mathcal {R}}_{w,12}&= {\mathcal {R}}_{w,21} = 0 ; \end{aligned}$$

25 $$\begin{aligned} {\mathcal {R}}_{w,13}&= {\mathcal {R}}_{w,31} = \frac{2}{h} \int _S {\text {d}}S \ (-y) ; \end{aligned}$$

26 $$\begin{aligned} {\mathcal {R}}_{w,23}&= {\mathcal {R}}_{w,32} = \frac{2}{h}\int _S {\text {d}}S \ x; \end{aligned}$$

27 $$\begin{aligned} {\mathcal {R}}_{w,33}&= \frac{2}{h} \int _S {\text {d}}S \ (x^2+y^2). \end{aligned}$$

Here, the off-diagonal components of the symmetric tensor $\mathcal {R}_w$ are explicit manifestations of hydrodynamic rotation–translation coupling for anisotropic particles, which vanish for particles with enough symmetry (Uspal et al. 2013).

Taking Eqs. (19) and (22) together, we find the same force balance that was obtained above, but with an explicit specification of the fluid and wall contributions:

28 $$\begin{aligned} \left( \begin{array}{c}\varvec{F} \\ T\end{array}\right) = -\eta {\mathcal {R}}\left( \begin{array}{c}\varvec{U}_{\text {p}} \\ \omega _{\text {p}}\end{array}\right) + \left( \begin{array}{c}\varvec{F}_0 \\ T_0 \end{array}\right) = \left( \begin{array}{c}\varvec{0} \\ 0 \end{array}\right) , \end{aligned}$$

with ${\mathcal {R}}= {\mathcal {R}}_f + {\mathcal {R}}_w$ the symmetric $3 \times 3$ overall resistance tensor. The accuracy of this equality is directly related to the accuracy of the assumptions underlying the Brinkman equation and the simple shear flow in the gaps. Notice that Eq. (28) is equivalent to Eq. (10), indicating that this result is not sensitive to the hydrodynamic model that one chooses for the hydrodynamic fluid–particle interaction, because of the overdamped nature of the system and the fact that there are no external force and torque acting on the particle.

### Numerical methods

The Stokes equation (1) or the Brinkman equation (12) can be solved numerically for a particle of any shape in the channel. In this work, we use the finite-element software COMSOL Multiphysics (COMSOL, Inc., Burlington, MA, USA) to find the flow field $\varvec{u}$ or $\bar{\varvec{u}}$. Using this solution, we can determine the forces acting on the particle and integrate its motion using a numerical scheme detailed below. The finite-element mesh is carefully chosen fine enough, such that doubling the minimum element size leads to a deviation in the force-free velocities of only 0.05%.

At a given particle position $(x_{\text {p}},y_{\text {p}})$ and orientation $\theta$, each column of the resistance tensor $\mathcal {R}$ is determined by imposing a single non-zero component of the particle velocities $(\varvec{U}_{\text {p}},\omega _{\text {p}})$, without external flow. The force and torque that determine the column of $\mathcal {R}$ are either found by numerically solving the Stokes equation (1) and integrating the obtained stress tensor via Eq. (4), or by solving the Brinkman equation (12) and integrating via Eqs. (18) and (23)–(27). Similarly, $\varvec{F}_0$ and $T_0$ are found by fixing the particle in the external flow, in either formalism.

With ${\mathcal {R}}, \varvec{F}_0$ and $T_0$ determined from the finite-element solutions, the force- and torque-free velocity $\varvec{U}_{\text {p}}(x,y,\theta )$ and angular velocity $\omega _{\text {p}}(x,y,\theta )$ are calculated from Eq. (28). Then, the particle position and orientation are integrated as

29 $$\begin{aligned} x(t + \varDelta t)&= x(t) + U_{p,x}(x(t),y(t),\theta (t)) \, \varDelta t \end{aligned}$$

30 $$\begin{aligned} y(t + \varDelta t)&= y(t) + U_{p,y}(x(t),y(t),\theta (t)) \, \varDelta t \end{aligned}$$

31 $$\begin{aligned} \theta (t + \varDelta t)&= \theta (t) + \omega _{\text {p}}(x(t),y(t),\theta (t)) \, \varDelta t, \end{aligned}$$

for some appropriately chosen time step $\varDelta t$. The inversion of $\mathcal {R}$ to obtain the solution of Eq. (28) and the numerical integration (29)–(31) are performed in MATLAB to obtain the position and orientation at the next time step, which are subsequently fed back into the finite-element calculations.

### Validity of the Brinkman equation

**Fig. 2:**
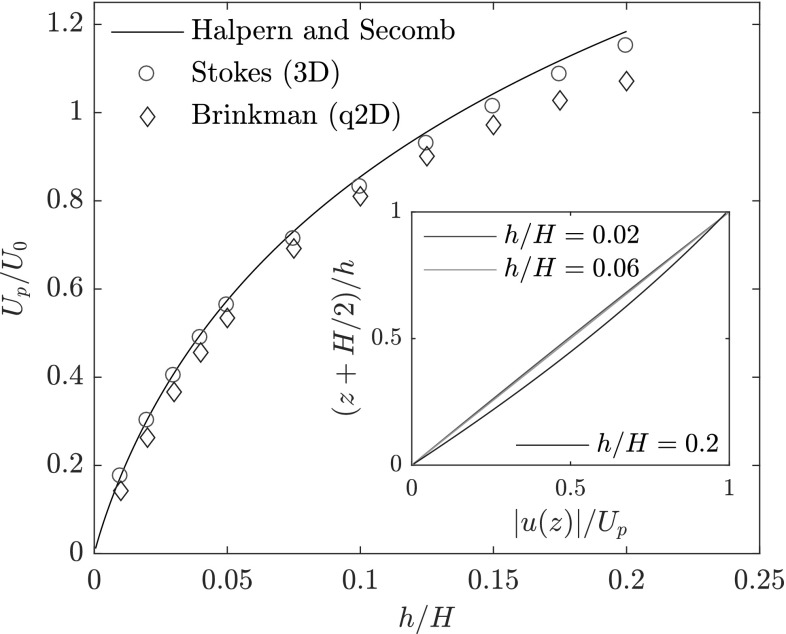
Terminal velocity of a rounded cylinder between two confining plates, as obtained by the from 3D finite-element method in the Stokes formalism (red) and 2D finite elements in the Brinkman formalism (blue), and the semi-analytical result from Halpern and Secomb (1991) (solid black line). The radius of the cylinder is $R/H = 1.79(1-2 h/H) + r_{\rm{c}}$, with radius of curvature $r_{\rm{c}} = (H-2h)/2$ for the rounded edges, where *H* and *h* are the channel and gap height, respectively. The inset shows the *z*-dependence of the flow field magnitude |*u*(*z*)| relative to the particle velocity $U_{\text {p}}$, at position $x=y=0$ in the thin gap between the bottom wall (at $z = -H/2$) and the particle disk surface (at $z = -H/2 + h$), for different gap heights *h*. (Colour figure online)

To test the validity of the Brinkman formalism described above, we compare with results obtained from three-dimensional analytical or numerical solutions of the Stokes equation. Here, we do this by considering the terminal velocity of a disk that is moving with the fluid between two infinite parallel plates (corresponding to the experimental setup but with *W* large compared to the disk size), for which a semi-analytical result by Halpern and Secomb exists (Halpern and Secomb 1991). The analysis in Halpern and Secomb (1991) concerns a cylindrical disk with rounded edges with a radius of curvature that is precisely half the height of the cylinder. In Fig. 2, we plot the particle velocity $U_{\text {p}}$, scaled by $U_0$, as a function of the gap height *h*/*H*. We compare the analytical solution to the results obtained from the solutions of the Stokes equation and of the Brinkman equation. We find good agreement between the three calculations, especially for smaller gaps. The deviations at larger gaps are expected, since the assumption of a simple shear flow and the quasi-2D character ceases to hold in this region. To test this, we plot, in the inset of Fig. 2, the magnitude of the flow field in the thin gap between the bottom wall at $z = -H/2$, where we have $|\varvec{u}| = 0$, and the disk surface at $z = -H/2 + h$, where $|\varvec{u}| = U_{\text {p}}$. We find that, for $h/H = 0.02$, the dependence of the flow field on the height *z* is linear, corresponding to simple Couette flow. Conversely, for $h/H = 0.2$, we find a deviation from the linear dependence, indicating that the simple shear flow assumption ceases to hold. The case of $h/H = 0.06$ corresponds to the experiments in this work, where, from Fig. 2, we see that the simple shear flow assumption is still valid.

It should be noticed that the rounding of cylinder edges is not taken into account in the quasi-2D calculations. This does not seem to influence the results strongly, since the results agree with the analytical and three-dimensional numerical results where the rounded edges are taken into account. An attempt to incorporate rounded edges, by making the gap height in our quasi-2D calculations position-dependent, did not improve the results significantly, but increased the computational time by a factor of two and was, therefore, not continued.

## Results

To test our numerical scheme for complex particle geometries, where analytical solutions are not available, we turn our attention to the situation described by Uspal et al. (2013). In Uspal et al. (2013) and in this work, dumbbell-shaped particles are produced in the channel using ‘continuous-flow lithography’. These dumbbell particles consist of two circular disks of radius $R_1$ and $R_2\le R_1$, respectively, and height $H - 2h$. The smaller disk has a fixed radius $R_2 = {18.75}\,\upmu {\text {m}}$, while the larger disk radius $R_1$ is varied. The disks are connected, at a fixed center-to-center distance $s = {62.5}\,\upmu {\text {m}}$, by a cuboid of width ${13.7}\,\upmu {\text {m}}$, which has the same height as the two disks. In the experiments, the dumbbell edges are not completely sharp but are rounded, with a rounding radius $r_{\text {c}}/H = 0.15$, as estimated from experimental images (Uspal et al. 2013). This detail is incorporated in the three-dimensional calculations, but not in the two-dimensional calculations. The particle geometry is depicted in Fig. 1b.

In this work, we focus on the aligning motion observed in Uspal et al. (2013), where asymmetric dumbbells oriented in the flow with the larger disk upstream. The orientation $\theta$ between the long axis of the dumbbell and the flow direction (see Fig. 1a) was shown to follow the equation:

32 $$\begin{aligned} \dot{\theta } = - \frac{1}{\tau } \sin \theta , \end{aligned}$$

where the characteristic timescale $\tau$ is dependent on the particle geometry. Specifically, we investigate here the dependence of $\tau$ on the ratio of the disk radii $R_1/R_2$. From Eq. (32), we see that $\tau$ can be obtained from a trajectory by considering the angular velocity at perpendicular orientation:

33 $$\begin{aligned} \tau = - \frac{1}{\dot{\theta } ( \theta = \pi /2)}. \end{aligned}$$

Hence, we can use the 3D and quasi-2D calculations described above to find the angular velocity of a particular dumbbell and obtain $\tau$ through Eq. (33).

**Fig. 3:**
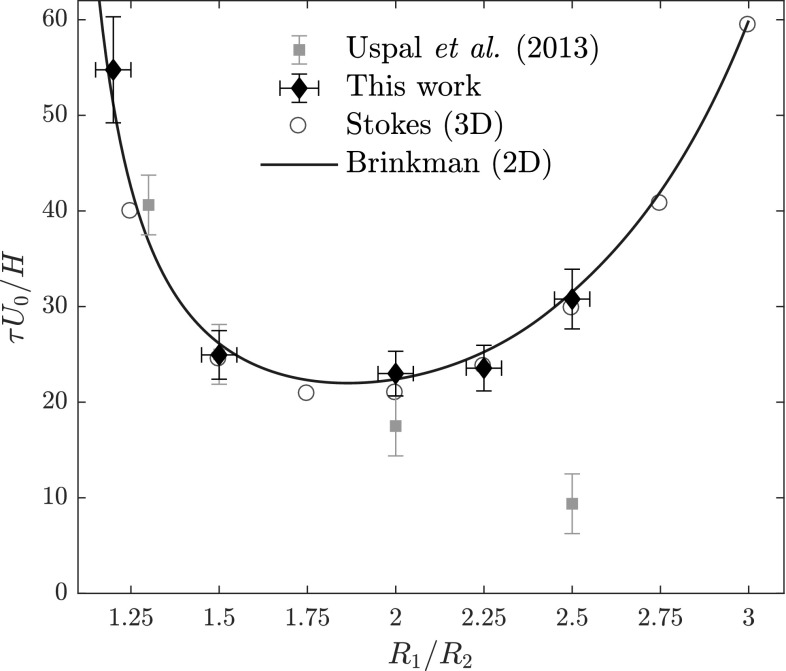
Characteristic reorientation time $\tau$ for dumbbell particles, as obtained from 3D finite-element method in the Stokes formalism (red) and 2D finite elements in the Brinkman formalism (blue), and experimental data from the previous experiments of Uspal et al. (2013) (green) and new experiments (black), as a function of $R_1/R_2$. (Colour figure online)

The results are shown in Fig. 3, where we plot $\tau$ against $R_1/R_2$. In green squares, we show the experimental data of Uspal et al. (2013); the red circles show the numerical data from our three-dimensional calculations, while the blue line shows the results from the quasi-two-dimensional calculations. First, we observe excellent agreement between the numerical results, which serves as another confirmation of the validity of our quasi-two-dimensional calculations. Second, we see that the results agree with the previous experimental data, with the exception of the data point of the most asymmetric size ratio $R_1/R_2 = 2.5$. Our calculations clearly show that a minimum in $\tau$ is expected around $R_1/R_2 \approx 1.9$, with $\tau$ increasing for larger $R_1/R_2$.

In contrast, the previous data show a strictly decreasing dependence of $\tau$ on $R_1/R_2$. Moreover, the theoretical curve that was fitted to the data in Uspal et al. (2013) was monotonically decreasing. This curve was derived considering an idealized situation of a pair of disks connected with a massless rod, which demonstrates the pitfalls of oversimplifying the geometry. However, we argue here that the minimum for $\tau$ that is predicted by our analysis is correct. In our calculations, as well as in the experiments, $R_1/R_2$ is varied by varying $R_1$, while keeping $R_2$ and the center-to-center distance *s* constant. As a result, when $R_1 \ge R_2 + s$, the shape becomes effectively just a single disk, which will not rotate at all ($\tau \rightarrow \infty$) due to symmetry (provided that it is still in the center of the channel). Therefore, when $R_1/R_2 \rightarrow \infty$, we should have $\tau \rightarrow \infty$ and hence a minimum in $\tau$. Note that the overlapping of the two disks is not incorporated in the theoretical results of Uspal et al. (2013).

To clear up this discrepancy, we performed a new set of experiments using an experimental setup that is almost identical to the setup in Uspal et al. (2013). Dumbbell particles were produced in a Hele–Shaw channel using continuous-flow lithography and their reorientation motion was tracked, as described in Appendix 1. In our experimental setup, the particle geometry and gap heights are taken to be approximately identical to those used in Uspal et al. (2013). Our measured reorientation times $\tau$ are shown by black diamonds in Fig. 3, where an excellent agreement with our numerical results is observed. The new data confirm the existence of a minimum in $\tau$ as a function of $R_1/R_2$. We stress that our numerical method does not rely on any adjustable parameter, and uses only the experimental geometry and flow rate as input.

**Fig. 4:**
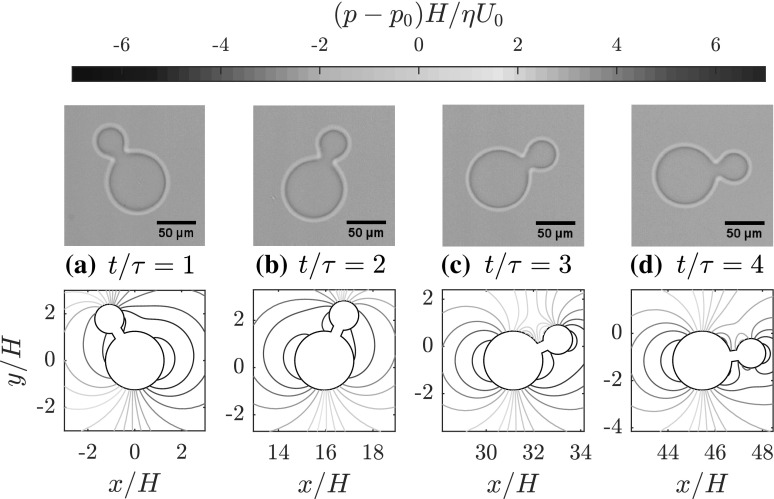
Experimental (top) and numerical (bottom) snapshots of the reorienting motion of a dumbbell with $R_1/R_2 =2$ in the microfluidic channel, at $t/\tau = 1$ (**a**), $t/\tau = 2$ (**b**), $t/\tau = 3$ (**c**), and $t/\tau = 4$ (**d**), where the initial orientation of the dumbbell is $\theta (0) = 5\pi /6$. We clearly observe that the dumbbell reorients with the larger disk upstream ($\theta =0$). Around the dumbbell, we show isobars of the disturbance pressure field created by the particle, as obtained from Eq. (12)

We have also calculated the complete angular trajectory of the dumbbell particles. Setting the time step to $\varDelta t /\tau = 0.2$, we integrated the position and orientation as described in Eqs. (29)–(31), starting from an initial orientation $\theta (t=0) = 5\pi /6$. Indeed, we observe that the dumbbells will align with the flow, such that the larger disk is upstream ($\theta = 0$). This is illustrated in Fig. 4, where we contrast experimental (top) and numerical (bottom) snapshots of the reorienting particle at times $t/\tau = 1$ (a), $t/\tau = 2$ (b), $t/\tau = 3$ (c), and $t/\tau = 4$ (d). We do not distinguish here between the $\tau$ values obtained from experiment and numerical calculations, since the two results are found to agree very well. Around the particle in the numerical snapshots of Fig. 4, we show isobars of the disturbance pressure field $p(x,y)-p_0(x)$ created by the particle, which are obtained from the Brinkman equation (12) with the force- and torque-free (angular) velocity imposed. Here, $p_0$ denotes the pressure field corresponding to an undisturbed external flow $U_0$ in the channel. The contours of $p(x,y)-p_0(x)$ show the dipolar nature of the disturbance flow. A clear impression of the particle motion may be obtained from the microscopic movie and animations found in the supplemental material.^3^

**Fig. 5:**
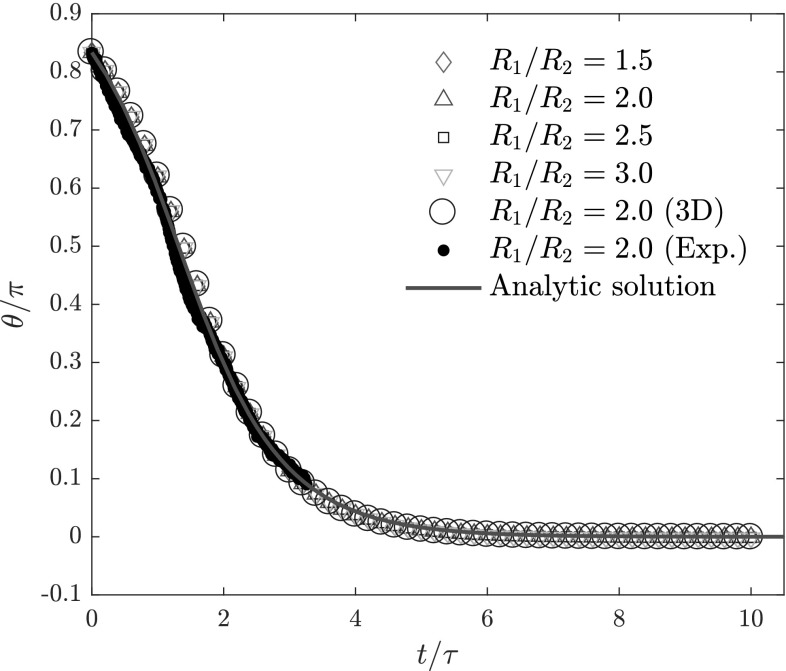
Orientation angle $\theta$ as a function of rescaled time $t/\tau$, with $\theta (t=0) = 5 \pi /6$, for dumbbells of varying $R_1/R_2$. The large blue circles show the trajectory obtained using the three-dimensional method, while the black dots show one particular trajectory obtained from the experiments, both for a dumbbell particle with $R_1/R_2 = 2$. Error bars for the experimental data are smaller than the black dots and hence omitted. The solid red line shows the analytical solution of Eq. (32)

The results for the complete angular trajectories are shown in Fig. 5. When time is rescaled by $\tau$, we find a data collapse of the numerical calculations (open symbols) on the analytical solution of Eq. (32) (solid red line). This collapse offers a strong confirmation that the orientation is, indeed, correctly described by Eq. (32). For $R_1/R_2 = 2.0$, we have also calculated a complete trajectory using the three-dimensional method. The results, shown by the large blue circles in Fig. 5, perfectly agrees with the quasi-two-dimensional results, a final confirmation of the accuracy of our quasi-two-dimensional calculations. Finally, we also show an experimentally obtained trajectory (black dots) and find good agreement with the numerical results. Discrepancies may be attributed to small variations in the channel height due to fabrication imperfections or dust particles in the prepolymer mixture.

## Summary and outlook

In conclusion, we have set up a combined theoretical and numerical framework that uses numerical solutions of either three-dimensional (Stokes) or quasi-two-dimensional (Brinkman) hydrodynamical equations, to calculate strongly confined particle motion in shallow microfluidic channels. Our method is not restricted to simplified shapes such as disks, but is able to handle any shape. The method is validated by comparing between analytical and three- and quasi-two-dimensional numerical calculations, which show excellent agreement. The two orders of magnitude of computational speedup that is offered by the quasi-two-dimensional description enable to fully resolve the particle trajectory in time.

Our method is applied to dumbbell particles, for which we have calculated the characteristic rotation time $\tau$ as a function of $R_1/R_2$, and found that, contrary to earlier findings, $\tau$ shows a minimum at $R_1/R_2 \simeq 1.9$. The existence of a minimum is confirmed by a new set of experiments. Moreover, we have calculated the angular motion as a function of time, and have shown that it agrees with the equation of motion derived earlier, and with the trajectories that are obtained from the experiments.

In future work, it will be interesting to further investigate the relation between the geometry and the trajectories of different particles, with the goal to possibly steer particles to different areas in the channel. Finally, our method is easily generalized to systems of multiple particles, which can offer a controlled setup to study hydrodynamic interaction between confined particles. Work in this direction is already under-way.

### Electronic supplementary material

Below is the link to the electronic supplementary material.

Supplementary material 1 (pdf 206 KB)

Supplementary material 2 (avi 15748 KB)

Supplementary material 3 (avi 3411 KB)

Supplementary material 4 (avi 13049 KB)

Supplementary material 5 (avi 8944 KB)

## Appendix 1: Materials and experimental methods

### Particle production and tracking

Polymeric microparticles are produced and observed with an experimental setup, similar to the one used by Uspal et al. (2013). Polydimethylsiloxane (PDMS, Sylgard^®^ 184, Dow Corning) and microfluidic devices are produced according to Dendukuri et al. (2008). A UV-crosslinking oligomer, poly-(ethyleneglycol) diacrylate (PEG-DA $M_n=700$, $\eta = 55 \times 10^{-3}\,{\text {Pa}}\,{\text {s}}$, Sigma-Aldrich), is mixed with a photoinitiator, hydroxy-2-methylpropiophenone, (Darocur^®^ 1173, Sigma-Aldrich), in a 19:1 volume ratio and the mixture is pumped through the microfluidic channel. The device, loaded with prepolymer, is mounted on the stage of a motorized Nikon Ti Eclipse inverted optical microscope. A photolithographic mask with well-defined shape is inserted between the UV light source and the microscope objective. Mask designs are made in Wolfram Mathematica^®^ and post-processed in Dassault Systémes’ DraftSight^®^.

Microparticles are produced by shining a 100 ms pulse of UV light through the mask onto the channel, thus confining photopolymerization to a discrete part of the prepolymer mixture. Oxygen, diffusing through the PDMS walls of the device, inhibits crosslinking (Dendukuri et al. 2008). This ensures the formation of two lubrication layers, which separate the particle from the *z*-walls, thus preventing sticking.

The microparticle is set in motion by applying a pressure drop $\Delta p$ across the channel and tracked by moving the automated stage in a stepwise manner. Since two different microfluidic devices with differing hydraulic resistances were used, the applied pressure differs depending on the device used—$\Delta p_1=0.10\pm 0.01 \,{\text {psig}}$ and $\Delta p_2=0.15\pm 0.01 \,{\text {psig}}$. Both pressure drops produce a flow with $U_0\approx {50}\,\upmu {\text {m}}\,{\text {s}}^{-1}$ in the respective microfluidic device. The experimental uncertainty in determining $U_0$ is around 10%.^4^

Particle positions and orientations are extracted from the acquired movie using a custom-written MATLAB script, which employs circular Hough transforms to identify the particle shape in each frame. The script utilizes MATLAB’s Bio-Formats package (Linkert et al. 2010) and the calcCircle tool.

### Channel geometry and determination

Both used devices feature a microfluidic channel of length ($L=11.54\pm 0.01\,{\text {cm}}$) and width ($W=515\pm 2\,\upmu {\text {m}}$) determined via optical microscopy. The channel height ($H=30\pm 1\,\upmu {\text {m}}$) is inferred from fluorescent particle tracking (Uspal et al. 2013), and verified with optical and scanning electron microscopy by cutting the device and looking at the cross section. Briefly, fluorescently labelled polystyrene beads with an average diameter of $1.89\,\upmu {\text {m}}$ are introduced in the prepolymer mixture. After focussing in the mid-plane of the channel, the beads are tracked, as the mixture is pumped through the device. Due to optical depth of focus and bead size, a normal distribution of particle velocities is obtained. The mean of the distribution is taken as $U_0$ and the height of the channel is calculated via Eq. 11. Particle height ($H_\text {p}=25.7 \pm 1.4\, \upmu {\text {m}}$) is measured via optical microscopy and is used to calculate the lubrication layer thickness ($h=(H-H_\text {p})/2=2.2 \pm 0.9\, \upmu {\text {m}}$).

## Appendix 2: Lorentz reciprocal theorem for Brinkman flow

In this appendix, we derive a version of the Lorentz reciprocal theorem for solutions of the Brinkman equation. Let $\bar{\varvec{u}}$ and $\bar{\varvec{u}}'$ be two solutions to the Brinkman equation in a two-dimensional fluid domain *A* with boundary $\partial A$, with different boundary conditions. These solutions have corresponding stress tensors $\bar{\sigma }$ and $\bar{\sigma }'$, defined as follows:

34 $$\begin{aligned} \bar{\sigma }_{ij} = - \bar{p} \delta _{ij} + \eta H (\partial _i \bar{u}_j + \partial _j \bar{u}_i), \end{aligned}$$

and similarly for $\bar{\sigma }'$. With this definition, we may write the Brinkman equation (12) as follows:

35 $$\begin{aligned} \nabla _{\text {2D}} \cdot \bar{\sigma } = \frac{12 \eta }{H} \bar{\varvec{u}}, \quad \nabla _{\text {2D}} \cdot \bar{\varvec{u}} = 0. \end{aligned}$$

Let us follow the same steps as in the derivation of the original Lorentz reciprocal theorem (Kim and Karrila 1991). First, consider the quantity $\bar{u}'_i(\partial _j \bar{\sigma }_{ij})$, which can be rewritten as follows:

36 $$\begin{aligned} \bar{u}'_i(\partial _j \bar{\sigma }_{ij})&= \partial _j(\bar{u}'_i \bar{\sigma }_{ij}) - \bar{\sigma }_{ij} \partial _j \bar{u}'_i = \partial _j(\bar{u}'_i \bar{\sigma }_{ij}) \nonumber \\&\quad - (-\bar{p} \delta _{ij} + \eta (\partial _{i} \bar{u}_{j}+ \partial _j \bar{u}_i))\partial _j\bar{u}'_i \nonumber \\&=\partial _j(\bar{u}'_i \bar{\sigma }_{ij}) - \eta (\partial _{i} \bar{u}_{j}+ \partial _j \bar{u}_i)\partial _j\bar{u}'_i, \end{aligned}$$

where the term involving $\bar{p}$ drops out due to the incompressibility constraint $\partial _i \bar{u}'_i = 0$. Interchanging $\bar{\varvec{u}}$ and $\bar{\varvec{u}}'$ and subtracting give us

37 $$\begin{aligned} \bar{u}'_i (\partial _j \bar{\sigma }_{ij}) - \bar{u}_i(\partial _j \bar{\sigma }'_{ij}) = \partial _j(\bar{u}'_i \bar{\sigma }_{ij} - \bar{u}_i \bar{\sigma }'_{ij}). \end{aligned}$$

For solutions of Stokes flow, both terms of the left-hand side of Eq. (37) vanish identically leading to the Lorentz reciprocal theorem. For solutions of the Brinkman equation, the left-hand side of Eq. (37) vanishes, because when Eq. (35) is applied to it, we find

38 $$\begin{aligned} \bar{u}'_i \frac{12 \eta }{H} \bar{u}_i - \bar{u}_i \frac{12 \eta }{H} \bar{u}'_i = 0, \end{aligned}$$

such that Eq. (37) reads

39 $$\begin{aligned} \nabla _{\text {2D}} \cdot (\bar{\varvec{u}}' \cdot \bar{\sigma } - \bar{\varvec{u}} \cdot \bar{\sigma }') = 0. \end{aligned}$$

Thus, using the two-dimensional divergence theorem, we obtain the Lorentz reciprocal theorem for Brinkman flow:

40 $$\begin{aligned} \oint _{\partial A} {\text {d}}s \bar{u} \cdot \bar{\sigma }' \cdot \varvec{n} = \oint _{\partial A} {\text {d}}s \bar{u}' \cdot \bar{\sigma } \cdot \varvec{n}, \end{aligned}$$

where $\partial A$ denotes the one-dimensional boundary of the two-dimensional fluid domain *A*, where $\bar{\varvec{u}}$ and $\bar{\varvec{u}}'$ are defined and solve the Brinkman equation. Using this relation, symmetry properties of the resistance tensor $\mathcal {R}_f$ defined in Eq. (19) for particles in Hele–Shaw cells may be proven, see Appendix 3.

## Appendix 3: Fluid resistance tensor from the Brinkman equation

In this appendix, we show explicitly that the force $\varvec{F}_f$ and torque $T_f$ exerted on the particle by the (quasi-)two-dimensional Brinkman fluid are related to the particle velocity and angular velocity in terms of a $3 \times 3$ resistance tensor $\mathcal {R}_f$. Inspired by the decompositions into sub-solutions of the original Stokes problem in Sect. 2.1, we exploit the linearity of the Brinkman equation to write the solutions as the superpositions $\bar{\varvec{u}} = \bar{\varvec{u}}_0 + \bar{\varvec{u}}' + \bar{\varvec{u}}''$ and $\bar{p} = \bar{p}_0 + \bar{p}' + \bar{p}''$, where $(\bar{p}_0,\bar{\varvec{u}}_0)$, $(\bar{p}',\bar{\varvec{u}}')$ and $(\bar{p}'',\bar{\varvec{u}}'')$ are solutions to the Brinkman equation (12) with boundary conditions:

41 $$\begin{aligned}&\bar{\varvec{u}}_0\big |_{\text {walls}} = 0, \quad \bar{\varvec{u}}_0\big |_{\text {inlet}} = U_0 \hat{\varvec{x}}, \quad \bar{\varvec{u}}_0\big |_{\varvec{r} \in \partial \bar{S}_{\text {p}}} = 0; \end{aligned}$$

42 $$\begin{aligned}&\bar{\varvec{u}}'\big |_{\text {walls}} = 0, \, \quad \bar{\varvec{u}}'\big |_{\text {inlet}} = 0, \qquad \ \, \bar{\varvec{u}}'\big |_{\varvec{r} \in \partial \bar{S}_{\text {p}}} = \varvec{U}_{\text {p}}; \end{aligned}$$

43 $$\begin{aligned}&\bar{\varvec{u}}''\big |_{\text {walls}} = 0, \quad \bar{\varvec{u}}''\big |_{\text {inlet}} = 0, \end{aligned}$$

44 $$\begin{aligned}&\bar{\varvec{u}}''\big |_{\varvec{r} \in \partial \bar{S}_{\text {p}}} = \omega _{\text {p}}(-(y-y_{\text {p}}),x-x_{\text {p}}), \end{aligned}$$

respectively. Here, $\bar{\varvec{u}}_0$ corresponds to the situation in which the particle is stationary subject to the imposed external flow, while $\bar{\varvec{u}}'$ and $\bar{\varvec{u}}''$ correspond to the situation where the particle is translating and rotating, respectively, in the absence of an imposed flow. The stress tensor of Eq. (16), being linear in the fields $\bar{\varvec{u}}$ and $\bar{p}$, admits a similar decomposition $\bar{\sigma } = \bar{\sigma }_0 + \bar{\sigma }' + \bar{\sigma }''$, which is then inherited by the force $\varvec{F}_f$ and torque $T_f$ via Eq. (18). While the force and torque on the stationary particle follow directly from Eq. (18) by substituting $\bar{\sigma }_0$ for $\bar{\sigma }$, let us consider the contributions from $\bar{\sigma }'$ and $\bar{\sigma }''$ in more detail.

### Translation

The linearity of the Brinkman equation allows for a factorization:

45 $$\begin{aligned} \bar{u}'_i = \bar{\mathcal{U}}'_{ij} U_{p,j}, \quad \bar{p}' = \eta \bar{\mathcal {P}}'_{ij} U_{p,j}, \end{aligned}$$

where the tensor fields $(\bar{\mathcal {U}}',\bar{\mathcal {P}}')$ obey

46 $$\begin{aligned}&- \partial _i \bar{\mathcal {P}}'_j + \partial _k^2 \bar{\mathcal {U}}'_{ij} -\frac{12 }{H} \bar{\mathcal {U}}'_{ij} = 0, \quad \partial _i {\mathcal {U}}'_{ij} = 0, \end{aligned}$$

47 $$\begin{aligned}&\bar{\mathcal {U}}'_{ij}\big |_{\text {walls}} = \bar{\mathcal {U}}'_{ij}\big |_{\text {inlet}} = 0, \quad \bar{\mathcal {U}}_{ij}'\big |_{\varvec{r} \in \partial \bar{S}_{\text {p}}} = \delta _{ij}, \end{aligned}$$

which lead to the original Brinkman equation for $(\bar{\varvec{u}}',\bar{p}')$ by contracting with $\varvec{U}_{\text {p}}$. In turn, the fields $(\bar{\mathcal {U}}',\bar{\mathcal {P}}')$ define a tensor

48 $$\begin{aligned} \bar{\varSigma }'_{ijk} = - \delta _{ij} \bar{\mathcal {P}}'_k + \partial _i \bar{\mathcal {U}}'_{jk} + \partial _j \bar{\mathcal {U}}'_{ik}, \end{aligned}$$

which is related to the stress tensor by $\bar{\sigma }'_{ij} = \eta \bar{\varSigma }'_{ijk} (U_{\text {p}})_k$. Next, we define

49 $$\begin{aligned} \bar{K}_{ik}&= - \oint _{\partial \bar{S}_{\text {p}}} {\text {d}}S (\bar{\varSigma }'_{ijk} n_j), \end{aligned}$$

50 $$\begin{aligned} \bar{C}'_{k}&= -\oint _{\partial \bar{S}_{\text {p}}} {\text {d}}S \bigg ( (x-x_{\text {p}}) (\bar{\varSigma }'_{y j k} n_j) - (y-y_{\text {p}}) (\bar{\varSigma }'_{x j k} n_j) \bigg ), \end{aligned}$$

such that the hydrodynamic force and torque that the two-dimensional fluid exerts on the translating particle are expressed as follows:

51 $$\begin{aligned} F'_{f,i} = - \eta \bar{K}_{ij} {U}_{p,j}, \quad T'_f = - \eta \bar{C}'_j {U}_{p,j} \ . \end{aligned}$$

Similar to the derivation for the three-dimensional resistance tensor, we can prove that the $2 \times 2$ tensor *K* is symmetric, using a version of the Lorentz reciprocal theorem that is proven in Appendix 2. Consider the two problems where the particle is either translating with velocity $\varvec{U}$ or $\tilde{\varvec{U}}$ through the channel without external flow, with corresponding flow fields $\bar{\varvec{u}}$ and $\tilde{\bar{\varvec{u}}}$ that are solutions to the Brinkman equation with boundary conditions (42). Applying the Brinkman version of the reciprocal theorem, we find

52 $$\begin{aligned}&\oint _{\partial \bar{S}_{\text {p}}} {\text {d}}s \bar{\varvec{u}} \cdot \tilde{\bar{\sigma }} \cdot \varvec{n} + \oint _{\text {w,i,o}} {\text {d}}s \bar{\varvec{u}} \cdot \tilde{\bar{\sigma }} \cdot \varvec{n} = \nonumber \\&\oint _{\partial \bar{S}_{\text {p}}} {\text {d}}s \tilde{\bar{\varvec{u}}} \cdot {\bar{\sigma }} \cdot \varvec{n} + \oint _{\text {w,i,o}} {\text {d}}s \tilde{\bar{\varvec{u}}} \cdot {\bar{\sigma }} \cdot \varvec{n}, \end{aligned}$$

where the integration over ‘$\text {w,i,o}$’ is carried out over the (projected) sidewalls, the inlet and the outlet. However, since both flow fields vanish on the side walls according to Eq. (42), these integrals vanish identically. Using the no-slip boundary condition on the particle boundary $\partial \bar{S}_{\text {p}}$ and definitions of Eq. (18), we find

53 $$\begin{aligned} \tilde{\varvec{U}} \cdot \varvec{F}_f = {\varvec{U}} \cdot \tilde{\varvec{F}}_f, \end{aligned}$$

which using Eq. (51) may be written as follows:

54 $$\begin{aligned} \tilde{U}_i \bar{K}_{ij} U_j = U_i \bar{K}_{ij} \tilde{U}_j = \tilde{U}_i \bar{K}_{ji} U_j. \end{aligned}$$

Now, since $\varvec{U}$ and $\tilde{\varvec{U}}$ are completely arbitrary, we conclude that $\bar{K}$ is symmetric: $\bar{K}_{ij} = \bar{K}_{ji}$.

### Rotation

Similarly, we factorize the angular velocity $\omega _{\text {p}}$ from the solution $\bar{\varvec{u}}''$:

55 $$\begin{aligned} \bar{u}''_i = \bar{\mathcal {U}}''_{i} \omega _{\text {p}}, \quad \bar{p} = \eta \bar{\mathcal {P}}'' \omega _{\text {p}}, \end{aligned}$$

with (tensor-)fields that obey

56 $$\begin{aligned}&- \partial _i \bar{\mathcal {P}}'' + \partial _k^2 \bar{\mathcal {U}}''_{i} -\frac{12 }{H} \bar{\mathcal {U}}''_{i} = 0, \quad \partial _i {\mathcal {U}}''_{i} = 0, \end{aligned}$$

57 $$\begin{aligned}&\bar{\mathcal {U}}''_{i}\big |_{\text {walls}} = \bar{\mathcal {U}}''_{i}\big |_{\text {inlet}} = 0, \quad \bar{\mathcal {U}}''_i\big |_{(x,y) \in \partial \bar{S}_{\text {p}}} = -y \delta _{ix } + x \delta _{i y}, \end{aligned}$$

which, as usual, lead to the original Brinkman equation for $\bar{\varvec{u}}''$ with boundary conditions (44) by multiplying with $\omega _{\text {p}}$. Moreover, we define the tensor $\bar{\varSigma }''_{ij}$, similar to Eq. (48), which has the property $\bar{\sigma }_{ij}'' = \bar{\varSigma }''_{ij} \omega _{\text {p}}$. Note that the fields $\bar{\mathcal {U}}'',\bar{\mathcal {P}}''$ and $\bar{\varSigma }''$ have dimensions of length of one power higher than their single-primed counterparts ${\mathcal {U}}', {\mathcal {P}}'$ and $\varSigma '$, while they are of one tensor rank lower, the latter following from the fact that we have only one rotational degree of freedom in two dimensions. To proceed, we define

58 $$\begin{aligned} \bar{C}''_{i}&= - \oint _{\partial \bar{S}_{\text {p}}} {\text {d}}S (\bar{\varSigma }''_{ij} n_j), \end{aligned}$$

59 $$\begin{aligned} \bar{\varOmega }&= -\oint _{\partial \bar{S}_{\text {p}}} {\text {d}}S (x-x_{\text {p}})(\bar{\varSigma }''_{y j} n_j) - (y-y_{\text {p}}) (\bar{\varSigma }''_{x j} n_j), \end{aligned}$$

such that

60 $$\begin{aligned} \varvec{F}''_{f,i} = -\eta \bar{C}''_i \omega _{\text {p}} , \quad T''_f = -\eta \bar{\varOmega }\omega _{\text {p}}. \end{aligned}$$

Similar to proving that $\bar{K}$ is symmetric, we can use the Lorentz reciprocal theorem of Appendix 2 to prove that, in fact, $C'_i = C''_i$, but we leave this to the reader.

### Complete particle motion

Taking together the contributions from the sub-solutions $\bar{\varvec{u}}_0, \bar{\varvec{u}}'$ and $\bar{\varvec{u}}''$, we find that the force and torque on the moving particle exerted by the two-dimensional fluid can be written as follows:

61 $$\begin{aligned} \left( \begin{array}{c}\varvec{F} \\ T\end{array}\right) _f = -\eta {\mathcal {R}}_f \left( \begin{array}{c}\varvec{U}_{\text {p}} \\ \omega _{\text {p}} \end{array}\right) + \left( \begin{array}{c}\varvec{F}_0 \\ T_0 \end{array}\right) , \end{aligned}$$

where the components of the $3\times 3$ resistance tensor $\mathcal {R}_f$ are given by the following:

62 $$\begin{aligned} {\mathcal {R}}_f = \left( \begin{array}{ccc}\bar{K}_{11} &{} \bar{K}_{12} &{} \bar{C}_1 \\ \bar{K}_{12} &{} \bar{K}_{22} &{} \bar{C}_2 \\ \bar{C}_1 &{} \bar{C}_2 &{} \bar{\varOmega }\end{array}\right) , \end{aligned}$$

with $$C_i \equiv C_i' = C_i''$$.

## Footnotes

1 For the remainder of this text, $\varvec{F}$ and $\varvec{U}_{\text {p}}$ denote two-dimensional force and particle velocity, respectively.

2 This can only be the case if the particle is not also confined in the *y*-direction, which is the case in the experimental situation of interest.

3 See Supplemental Material for the animation and microscopic movie of a reorienting dumbbell particle.
