# Supplementary material for: Calculating the motion of highly confined, arbitrary-shaped particles in Hele–Shaw channels
Source: Microfluid Nanofluidics. 2018 Jul 12;22(8):77. doi: 10.1007/s10404-018-2092-y (PMC6394751; doi:10.1007/s10404-018-2092-y)
Supplement: Supplementary file 1 — Supplementary material 1 (pdf 206 KB) [file 10404_2018_2092_MOESM1_ESM.pdf]

Supplemental material: Calculating the motion of highly confined, arbitrary-shaped particles in Hele-Shaw channels

Bram Bet,[1,*] Rumen Georgiev,[2] William Uspal,[3]
Burak Eral,[2] René van Roij,[1] and Sela Samin[1]

[1]*Institute for Theoretical Physics, Center for Extreme Matter and Emergent Phenomena, Utrecht University, Princetonplein 5, 3584 CC Utrecht, The Netherlands*

[2]*Process and Energy Department, Delft University of Technology, Delft 2628 CD, The Netherlands*

[3]*Max Planck Institute for Intelligent Systems, Heisenbergstr. 3, 70569 Stuttgart, Germany*

(Dated: October 9, 2017)

* B.p.bet@uu.nl

I. DESCRIPTION OF THE MOVIES

Here, we give a short description of the experimentally-obtained movie and the animations provided as supplementary information. Definitions of the shape parameters, the channel dimensions and other parameters may be found in the main text.

M1 Movie 1 is obtained from microscopic images and shows a dumbbell particle with $R_1/R_2 = 2$ rotating towards a stable orientation with the large disk upstream, while translating in the positive x-direction (to the right), along with the externally imposed flow. The field of view of the microscope follows the particle in a stepwise manner.

A1 Animation 1 shows a reorienting dumbbell particle with $R_1/R_2 = 2$, as seen from a fixed (laboratory) frame. The color of the heat map indicates the magnitude of the flow field $\boldsymbol{u}$ in units of U_0.

A2 Animation 2 shows again a reorienting dumbbell particle with $R_1/R_2 = 2$, now seen from a frame co-moving with the particle. The arrows indicate the disturbance flow field $\bar{\boldsymbol{u}} - \bar{\boldsymbol{u}}_0$, where $\bar{\boldsymbol{u}}_0$ is the externally imposed flow field far away or in the absence of the particle. The colors of the arrows correspond to the magnitude $|\bar{\boldsymbol{u}} - \bar{\boldsymbol{u}}_0|$ of the disturbance flow field.

A3 Animation 3 shows again a reorienting dumbbell particle with $R_1/R_2 = 2$, seen from a frame co-moving with the particle, now shown with isobars of the disturbance pressure field $p - p_0$, in units of $\eta U_0/H$. Here, p_0 denotes the externally imposed pressure field far away from the particle.

II. DETERMINATION OF EXPERIMENTAL UNCERTAINTY

To calculate the experimental uncertainty in determining the scaled rotation time $\tilde{\tau} = \tau U_0/H$ and the time evolution of the particle orientation with respect to the flow $\theta\,(t)$, we carry out an error propagation analysis. In the general case of an experimental quantity F dependent on a series of experimentally-determined quantities $\{x_i\}$ with uncertainties $\{\sigma_{x_i}\}$, one calculates the uncertainty in F as:

$$\sigma_F = \sqrt{\sum_{\{x_i\}} \left(\frac{\partial R\,(\{x_i\})}{\partial x_i} \sigma_{x_i} \right)^2}. \tag{1}$$

A. Uncertainty in the particle thickness

The thickness of the particles, $H_\mathrm{p} = H - 2h$, is measured at the outlet, where they topple over. The particles are observed from the side and the distance between the edges of the top and bottom face is calculated. Due to uneven illumination of the faces and low contrast of the system, the edges appear thicker than one pixel, therefore a type B uncertainty should be taken into consideration. Type B uncertainty reflects the random nature of a process and any human bias in measuring it. Since most observed edges are roughly 5 pixels thick, a type B uncertainty σ_B of 3 pixels is estimated.

The finite spacial resolution of the microscope used suggests a type A uncertainty, as well, which is estimated to be the size of a single pixel at 40x magnification with 2-by-2 pixel binning – $\sigma_\mathrm{A} = 0.323\,\mu\mathrm{m/px}$. The total uncertainty in determining the position of a given edge is a product of σ_A and σ_B and amounts to roughly a micron.

To determine the uncertainty in the thickness, we use Eq. 1, in which $F(\{x_i\}) = H_\mathrm{p}(z_1, z_2) = z_1 - z_2$ and $\sigma_{z_1} = \sigma_{z_2} = 1\,\mu\mathrm{m}$:

$$\sigma_{H_\mathrm{p}} = \sigma_{z_1}\sqrt{2} = 1.4\,\mu\mathrm{m} \tag{2}$$

The thickness of 20 cylinders, produced in the microfluidic device, is measured. An average thickness $\langle H_\mathrm{p} \rangle = 25.7\,\mu\mathrm{m}$ and sample standard deviation $s = 0.3\,\mu\mathrm{m}$ is calculated. The minimum and maximum values are $H_\mathrm{p,min} = 25.0 \pm 1.4\,\mu\mathrm{m}$ and $H_\mathrm{p,max} = 26.1 \pm 1.4\,\mu\mathrm{m}$. Since the uncertainty in the measurements is greater than the sample standard deviation, we report $\langle H_\mathrm{p} \rangle = 25.7 \pm 1.4\,\mu\mathrm{m}$.

B. Uncertainty in the channel height

To determine the channel height using optical microscopy, the microfluidic chip is cut with a razor and the cross section is viewed. The type B uncertainty in this case is 2 pixels since the channel edges are sharper than those of PEG-DA particles. The type A uncertainty remains the size of a single pixel. Following the same approach like in the previous case, an uncertainty $\sigma_H = 2 \times 0.323\sqrt{2} = 0.9\,\mu\mathrm{m}$ is calculated.

C. Uncertainty in the average flow velocity

Obtaining the mean flow velocity requires fluorescent particle tracking in which a video of fluorescent beads flowing through the channel is recorded. The positions of a given particle in two consecutive frames are compared and the path length is calculated as their difference. Similarly to the uncertainties in channel and particle height, the uncertainty in the flow velocity scales linearly with the length-to-pixel ratio of the lens used and the uncertainty in determining the center of a given particle. The particle tracking algorithm uses a weighted centroid method to calculate the particle center, which has sub-pixel accuracy. We will make a non-conservative estimate and limit the type B uncertainty to half a pixel. Then, the uncertainty in the distance traveled by any particle over two consecutive frames is $\sigma_{\Delta x} = 0.323/2\sqrt{2} = 0.23\,\mu\mathrm{m}$. The frame rate of the movie is 20 FPS, therefore the lag between two consecutive frames is $t_\mathrm{f} = 0.05\,\mathrm{s}$. Applying Eq. 1 to the expression for the particle velocity $v_\mathrm{p} = \Delta x/t_\mathrm{f}$ yields:

$$\sigma_{v_\mathrm{p}} = \frac{\sigma_{\Delta x}}{t_\mathrm{f}} = 4.6\,\mu\mathrm{m\,s^{-1}} \tag{3}$$

D. Uncertainty in the disk position in a dumbbell

The uncertainty in determining where the center of a disk within a dumbbell lies is governed by the spacial resolution of the microscope and the accuracy of the numerical algorithm used to detect the disks. While tracking dumbbells, we use a 20x lens with 2-by-2 pixel binning, which results in a distance-to-pixel ratio of 0.650 µm/px. The circular Hough transform we use to detect the disks utilizes a weighted centroid method, which reports the coordinates of the disk center with a sub-pixel accuracy. However, the algorithm reports radii with a resolution of 1 pixel. Therefore, we estimate an uncertainty of 1 pixel or 0.650 µm in determining the disk coordinates.

E. Uncertainty in the shaft length in a dumbbell

Although the center-to-center distance of the two disks is fixed, there is both type A and type B uncertainty in its determination. We discussed the type A uncertainty in the previous section. The type B uncertainty stems from the randomness of the photopolymerization

reaction – different particles have slightly different shaft lengths. To estimate the uncertainty in the length of the shaft, we write the expression for it explicitly and apply Eq. 1 to it:

$$|\boldsymbol{O}(t)| = l = \sqrt{O_x^2(t) + O_y^2(t)} = \sqrt{\left(x_{\mathrm{S}}(t) - x_{\mathrm{B}}(t)\right)^2 + \left(y_{\mathrm{S}}(t) - y_{\mathrm{B}}(t)\right)^2}. \tag{4}$$

$$\begin{aligned}
\sigma_l &= \sqrt{\left(\frac{\partial l}{\partial x_{\mathrm{S}}}\sigma_{x_{\mathrm{S}}}\right)^2 + \left(\frac{\partial l}{\partial x_{\mathrm{B}}}\sigma_{x_{\mathrm{B}}}\right)^2 + \left(\frac{\partial l}{\partial y_{\mathrm{S}}}\sigma_{y_{\mathrm{S}}}\right)^2 + \left(\frac{\partial l}{\partial y_{\mathrm{B}}}\sigma_{y_{\mathrm{B}}}\right)^2} \\
&= \sqrt{\frac{2\left(x_{\mathrm{S}}(t) - x_{\mathrm{B}}(t)\right)^2 \sigma_{x_{\mathrm{S}}}^2}{l^2} + \frac{2\left(y_{\mathrm{S}}(t) - y_{\mathrm{B}}(t)\right)^2 \sigma_{y_{\mathrm{S}}}^2}{l^2}} \\
&= \sqrt{\frac{2\sigma_{x_{\mathrm{S}}}}{l^2} l^2} = \sigma_{x_{\mathrm{S}}}\sqrt{2} = 0.9\,\mu\mathrm{m}.
\end{aligned} \tag{5}$$

F. Uncertainty in the orientation angle

The orientation angle of the microparticle $\theta(t)$ is calculated from the vector $\boldsymbol{O}(t)$, which starts from the center of the big disk of a dumbbell and ends in the center of the small disk:

$$\theta(t) = \arccos\left(\frac{\boldsymbol{O}(t)\cdot\boldsymbol{e}_0}{|\boldsymbol{O}(t)||\boldsymbol{e}_0|}\right) = \arccos\left(\frac{O_x(t)}{|\boldsymbol{O}(t)|}\right) = \arccos\left(\frac{O_x(t)}{\sqrt{O_x^2(t) + O_y^2(t)}}\right), \tag{6}$$

where $\boldsymbol{e}_0$ is the unit vector aligned with the flow velocity. It is made sure the channel is leveled in such a way that the flow direction is parallel to the x-coordinate of the field of view. This reduces the dot product of the two vectors to the x-component of $\boldsymbol{O}(t)$. Applying Eq. 1 to Eq. 6 yields:

$$\begin{aligned}
\sigma_\theta &= \sqrt{\left(\frac{\partial \theta}{\partial x_{\mathrm{S}}}\sigma_{x_{\mathrm{S}}}\right)^2 + \left(\frac{\partial \theta}{\partial x_{\mathrm{B}}}\sigma_{x_{\mathrm{B}}}\right)^2 + \left(\frac{\partial \theta}{\partial y_{\mathrm{S}}}\sigma_{y_{\mathrm{S}}}\right)^2 + \left(\frac{\partial \theta}{\partial y_{\mathrm{B}}}\sigma_{y_{\mathrm{B}}}\right)^2} \\
&= \sqrt{2\frac{\sigma_{x_{\mathrm{S}}}^2}{l^2}} = \frac{\sigma_{x_{\mathrm{B}}}}{l}\sqrt{2} = 0.84^\circ.
\end{aligned} \tag{7}$$

We make a non-conservative estimate and round the uncertainty in the orientation angle up to 1°.

G. Uncertainty in the characteristic rotation time τ

The rotation time is easily accessible numerically via the rotation velocity at $\theta = \pi/2$. Experimentally, however, this is not a viable option because this would mean estimating τ

from only two micrographs with great uncertainty. To obtain a statistically-significant result experimentally, we fit the time evolution of the orientation angle to an analytical expression for $\theta(t)$. Solving Eq. 28 in the main text by making the substitution $\sin(\theta) = 2x/(1+x^2)$ yields:

$$-\frac{t}{\tau} = \ln\left(\frac{\tan(\theta/2)}{\tan(\theta_0/2)}\right). \tag{8}$$

Rearranging, we obtain the equation in linear form:

$$\ln(\tan(\theta/2)) = \ln(\tan(\theta_0/2)) - \frac{1}{\tau}t. \tag{9}$$

We calculate τ from the slope of a line fitted to a dataset $[t, \ln(\tan(\theta/2))]$. The estimated slope has an uncertainty since the data cannot be perfectly fitted to a line. The uncertainty in the slope b is inversely proportional to the square root of the number of data points N used in the linear regression:

$$\sigma_b = b\frac{1}{\sqrt{N-2}}\sqrt{\frac{1}{R^2} - 1}, \tag{10}$$

where R^2 reflects the goodness of the fit. In our experiments the relative uncertainty in the slope is less than 1.5% due to:

1. the great number of images we record – each experimental movie consists of at least 600 frames.

2. the high R^2 value – in all experiments $R^2 > 0.980$.

H. Uncertainty in the scaled rotation time $\tilde{\tau}$

Applying Eq. 1 to the scaled rotation time $\tilde{\tau} = \tau U_0/H$ yields:

$$\begin{aligned} \sigma_{\tilde{\tau}} &= \sqrt{\frac{U_0^2}{H^2}\sigma_\tau^2 + \frac{\tau^2}{H^2}\sigma_{U_0}^2 + \frac{U_0^2\tau^2}{H^4}\sigma_H^2} \\ &= \sqrt{\tilde{\tau}^2\frac{\sigma_\tau^2}{\tau^2} + \tilde{\tau}^2\frac{\sigma_{U_0}^2}{U_0^2} + \tilde{\tau}^2\frac{\sigma_H^2}{H^2}} \\ &= \tilde{\tau}\sqrt{\frac{\sigma_\tau^2}{\tau^2} + \frac{\sigma_{U_0}^2}{U_0^2} + \frac{\sigma_H^2}{H^2}} \end{aligned} \tag{11}$$

The three relative uncertainties are $\sigma_\tau/\tau \simeq 1\%$, $\sigma_{U_0}/U_0 \simeq 10\%$ and $\sigma_H/H = 3\%$. Filling in these numbers in Eq. 11 yields a relative uncertainty in $\tilde{\tau}$ of roughly 11% – the uncertainty is entirely dominated by the uncertainty in U_0.
